# Vaginal Microbiota Composition Correlates Between Pap Smear Microscopy and Next Generation Sequencing and Associates to Socioeconomic Status

**DOI:** 10.1038/s41598-019-44157-8

**Published:** 2019-05-23

**Authors:** Seppo Virtanen, Tiina Rantsi, Anni Virtanen, Kaisa Kervinen, Pekka Nieminen, Ilkka Kalliala, Anne Salonen

**Affiliations:** 1 Obstetrics and Gynecology, University of Helsinki and Helsinki University Hospital, Helsinki, Finland; 2 Department of Pathology, University of Helsinki and HUSLAB, Helsinki University Hospital, Helsinki, Finland; 3 Finnish Cancer Registry, Helsinki, Finland; 4 Human Microbiome Research Program, Faculty of Medicine, University of Helsinki, Helsinki, Finland; 5 Department of Surgery and Cancer, Imperial College London, London, UK

**Keywords:** Microbiome, Translational research

## Abstract

Recent research on vaginal microbiota relies on high throughput sequencing while microscopic methods have a long history in clinical use. We investigated the correspondence between microscopic findings of Pap smears and the vaginal microbiota composition determined by next generation sequencing among 50 asymptomatic women. Both methods produced coherent results regarding the distinction between *Lactobacillus*-dominant versus mixed microbiota, reassuring gynaecologists for the use of Pap smear or wet mount microscopy for rapid evaluation of vaginal bacteria as part of diagnosis. Cytologic findings identified women with bacterial vaginosis and revealed that cytolysis of vaginal epithelial cells is associated to *Lactobacillus crispatus*-dominated microbiota. Education and socio-economic status were associated to the vaginal microbiota variation. Our results highlight the importance of including socio-economic status as a co-factor in future vaginal microbiota studies.

## Introduction

Vaginal microbiota (VMB) and especially the presence of lactobacilli are important in maintaining the vaginal health and protecting the reproductive system from harmful organisms^1^. Based on molecular, culture-independent methods, the VMB can be clustered into five community state types (CSTs), of which four (CST I, CST II, CST III and CST V) are dominated by different species of *Lactobacillus* (*L*. *crispatus*, *L*. *gasseri*, *L*. *iners* and *L*. *jensenii*, respectively)^2^. Notably, all lactobacilli are not equal in their ability to maintain homeostasis in the vagina. *L*. *crispatus* appears as most stable and distinctive to a healthy state, whereas *L*. *iners* is found both in healthy women and those with dysbiosis and disease^3^, and its dominance relates to a higher risk of shifting into a non-*Lactobacillus*-dominated VMB^4^, i.e. CST IV. The CST IV refers to a mixed community enriched in anaerobic bacteria, such as *Gardnerella*, *Atopobium*, *Sneathia*, *Prevotella* or *Firmicutes* within *Lachnospiraceae* family that is characteristic for women with bacterial vaginosis (BV), but also for a subset of healthy women^1^. Hence, apart from the *L*. *crispatus*-dominated communities, it is difficult to distinguish the different variations of normality from an abnormal VMB.

Age, ethnicity, menstruation cycle, lifestyle habits, use of contraceptives, antibiotics and probiotics may have an impact on the VMB^2,5–7^. However, the associations between the microbiota, background variables, and clinical outcomes in different states of woman’s life are complex, and there is limited understanding on which intrinsic or external factors drive the community composition^8^. Behavioral factors, such as smoking^9,10^, sexual behavior^11–13^ and vaginal douching^14^ appear as risk factors for *Lactobacillus*-deficient VMB and BV. Ethnicity has impact on the composition of VMB also after controlling the confounding factors, such as sociodemographic, behavioral or environmental variables^2,15^. Studies directly investigating the impact of host socioeconomic or educational factors on VMB are rare.

In contrast to VMB research based on molecular studies and phenotypic characterization of bacterial isolates, the clinical diagnosis of vulvovaginal infections relies largely on descriptive and microscopic investigations or just on visual appearance of vaginal discharge. Microscopic examination of wet mount or Gram-stained vaginal discharge preparations are the current gold standard methods for the diagnosis of BV as part of the Amsel criteria^16^ or the sole component of the Nugent score^17^, respectively. Light microscopy of Papanicolaou-stained vaginal smears (Pap smears) used in cytological screening for early detection of cervical intraepithelial lesions provides information on both bacteria and host cells, and have been shown to provide diagnostic accuracy for BV that is comparable to the Amsel criteria and Nugent score^18^. Apart from BV, the diagnosis of less well known aerobic vaginitis (AV) is also based on microscopy-based scoring^19,20^. While these scores are used for clinical phenotyping of the study subjects in most molecular VMB studies, direct comparisons between the microscopic and molecular readouts have so far been limited to few studies that have focused on BV and compared Gram-stained bacterial morphotypes to selected bacterial groups^21,22^, or to community-wide microbiota analysis in a heterogeneous group of ethnically diverse, symptomatic women^23^.

Our objective was to evaluate the correspondence between the microscopic findings on Pap smear samples and the phylogenetic composition of VMB analyzed by 16S rRNA gene amplicon sequencing among unselected reproductive-aged women, and to evaluate the impact of individual background variables on the VMB composition.

## Results

### Description of the study cohort

We sampled 50 consecutive unselected non-pregnant Caucasian women aged 25–45 attending population-based organized cervical cancer screening in Helsinki, Finland. The mean age of the women was 32.6 years (median 29.5; SD 7.1; range 24–45). Further characteristics of the study population are discussed in the context of their relationship with the VMB.

### 16S rRNA gene sequencing results

**Figure 1:**
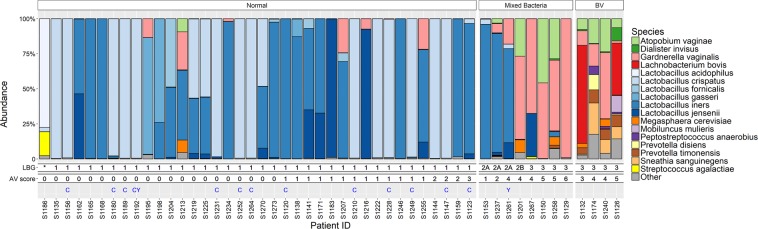
Sequencing results compared to the bacterial and other microscopic findings in the Pap smears. The colored bars represent sequencing-based bacterial composition for each subject, other features are based on microscopy of Pap smears. The subjects are grouped based on the microscopy as follows: Group ‘Normal’ represents usual rod-shaped bacteria, ‘Mixed Bacteria’ represents atypical or mixed bacteria without clue cells and ‘BV’ represents subjects with clue cells. *Lactobacillus* grade (LBG) and modified aerobic vaginitis score (AV) can be found below the bars. Presence of cytolysis (C) and yeast (Y) in the smears are indicated by letters. *Pap smear did not contain enough bacteria for LBG classification.

Altogether 41 (82.0%) of the 50 women had *Lactobacillus*-dominated VMB. The most dominant species were *L*. *iners* in 19/50 (38%; mean abundance 80.6% when dominant) and *L*. *crispatus* in 17/50 (34%; mean abundance 91.0% when dominant) women (Fig. 1). Other dominant *Lactobacillus* species were *L*. *jensenii* (2/50, 4%), *L*. *gasseri* (2/50, 4%), and *L*. *acidophilus* (1/50, 2%). Non-*Lactobacillus*-dominated VMB was present in nine (18%) women. In the non-*Lactobacillus*-dominated group (9/50), *Gardnerella vaginalis* was the dominant species in 6 women (66.7%; mean abundance 62.9% when dominant). Among all 50 women, the bacteria with top relative abundances were *L*. *iners* (33.8%), *L*. *crispatus* (31.2%), *G*. *vaginalis* (10.5%), *L*. *jensenii* (7.6%), *L*. *gasseri* (3.4%), *Atopobium vaginae* (3.4%), and *Lachnobacterium bovis* (2.2%). Altogether 37 different bacterial species were found, representing 22 genera (Supplementary Table S1).

### Pap smear results

In microscopy, the vaginal smears of 37 women with only rod-shaped *Lactobacillus*-type bacteria visible in their smears were defined ‘normal’ with respect to bacterial flora. The samples of four women showed signs of BV, defined by the presence of clue cells^24^, and the smears of eight women showed an altered bacterial flora without the presence of clue cells, ‘mixed bacteria’ (Fig. 1). All the samples were also graded with *Lactobacillus* Grade (LBG) independently from the previous classification criteria. All the samples graded LBG I were also defined ‘normal’. All the women with the signs of BV in their vaginal smears were classified as LBG III, whereas women with ‘mixed bacteria’ were classified as LBG IIa to LBG III (Fig. 1), depending on the ratio of rod-shaped bacteria to other types of bacteria. In one smear, hardly any bacteria could be identified in the microscopy and it could not graded with LGB.

For four subjects, the modified aerobic vaginitis (AV)-score was 5–6, corresponding to moderate AV in the original classification^19^. Three of these subjects showed signs of mixed bacteria and one of BV (Fig. 1). Cytolysis was detected in 13, and yeast cells or thread-like hyphae in two samples. Two women out of the 50 had abnormal cytological findings, one of LSIL (low-grade squamous intraepithelial lesion) and another of ASC-US (atypical squamous cells of undetermined significance). All the smears were satisfactory for cytological evaluation.

### Correspondence between bacterial findings in the Pap smears and the sequencing results

**Figure 2:**
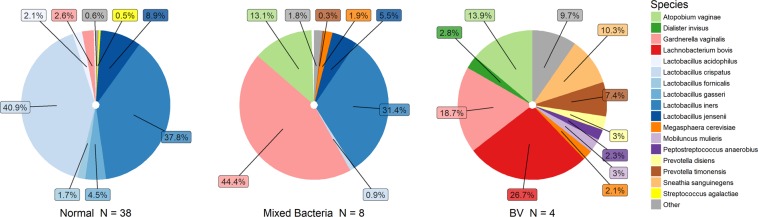
Average vaginal microbiota composition according to grouping based on microscopic examination of the Pap smears. The dominant species in different groups were *L*. *crispatus* for ‘normal’ (40.9%), *G*. *vaginalis* for ‘mixed bacteria’ (44.4%) and *L*. *bovis* for ‘BV’ (26.7%). The ‘BV’ group is very heterogenous and individual microbiota compositions can be seen in Fig. 1.

Figure 2 shows the average VMB composition measured through 16S rRNA gene sequencing in the three groups defined by microscopy; ‘normal’, ‘mixed bacteria’ and ‘BV’. Samples classified as LBG I i.e. ‘normal’ in microscopy had characteristic *Lactobacillus* dominance compared to those categorized as LBG III (Fig. 1). The *Lactobacillus*-deficient LBG III group was associated most strongly with the presence of *Mobiluncus mulieris*, *L*. *bovis*, and *G*. *vaginalis* (full list of associated species can be found in Supplementary Table S2), and had significantly higher species diversity than LBG I, which was associated with the abundance of *L*. *crispatus* and *L*. *iners*. The higher the lactobacilli count observed in microscopy, the more abundant were *Lactobacillus* species in sequencing (Supplementary Table S2). However, *L*. *iners* was not significantly associated with the lactobacilli count in microscopy, and *L*. *acidophilus* was not seen at all in microscopy (dominant only in one sample) (Fig. 1). As expected, samples with BV (clue cells) had significantly higher species diversity, and significantly less *L*. *crispatus* and *L*. *iners* than other samples based on sequencing. The most dominant species within the diverse ‘BV’ group was *L*. *bovis* and the group was significantly associated also to numerous other species, e.g. *M*. *mulieris*, *Prevotella timonensis*, *A*. *vaginae*, and *Sneathia sanguinegens* (Supplementary Table S2). The ‘mixed bacteria’-group dominated by *G*. *vaginalis* seemed to be a grey area between the ‘normal’ and ‘BV’ as some samples were dominated by *L*. *iners* instead of *G*. *vaginalis* or the combination of *G*. *vaginalis* and *A*. *vaginae* (Fig. 1).

Women with modified AV-score 4–5 had significantly more *G*. *vaginalis* and less *L*. *crispatus* than patients with modified AV-score 0 (p < 0.05). Microbiota diversity correlated positively with the modified AV-score from 0 to 5 (p < 0.05), while in the single woman with modified AV-score of 6, indicating more severe vaginitis, the diversity was extremely low due to the sole dominance of *G*. *vaginalis* (99%).

### Correspondence between other microscopic findings in the Pap smear samples and the sequencing results

The samples with cytolysis, defined as disintegration of intermediate epithelial cells, were significantly enriched for *L*. *crispatus* and depleted for *A*. *vaginae*, *G*. *vaginalis*, and *L*. *iners* compared to samples without cytolysis (Supplementary Table S2). Altogether 11/13 (85%) subjects with cytolysis had *L*. *crispatus*-dominated VMB and 11/17 (65%) of those with *L*. *crispatus*-dominated VMB had cytolysis. The remaining two patients with cytolysis had *L*. *iners* dominated VMB. Blood in the Pap smear sample was not associated with the changes in diversity or composition of the microbiota. The number of leucocytes in the microscopy was positively associated with the abundance of *Pseudomonas veronii* (Supplementary Table S2). Due to anecdotal numbers, atypical cytology and yeast in the Pap smears were not tested for associations with the microbiota. Human papilloma virus (HPV) positivity (7/50, 14% positive) was not associated to any microbiota feature in this cohort.

### Relationships between microbiological and demographic characteristics in the cohort

Self-reported demographic and lifestyle variables are summarized in Supplementary Table S3. To provide a simplified overview of the association between the VMB and these variables, we categorized the VMB into three major VMB clusters. These VMBs consisted predominantly of *L*. *crispatus* (n = 17 [34%], CST I), *L*. *iners* (n = 20 [40%], CST III) or diverse non-*Lactobacillus* species (n = 9 [18%], CST IV). Women with *L*. *crispatus* dominated VMB were younger than women in the other two VMB clusters (mean 30.3 years [range 25–45] vs. 35.2 years [range 25–45], p = 0.02). *L*. *crispatus* dominated VMB was also associated with higher education (p < 0.001 for trend). Women with *Lactobacillus* deficient VMB were more likely single or divorced than married or cohabiting (77.8% vs. 22.2%, p = 0.03) and had more often a history of fertility treatment, compared to women with *Lactobacillus*-dominated VMB (33.3% vs. 4.9%, p = 0.03).

**Figure 3:**
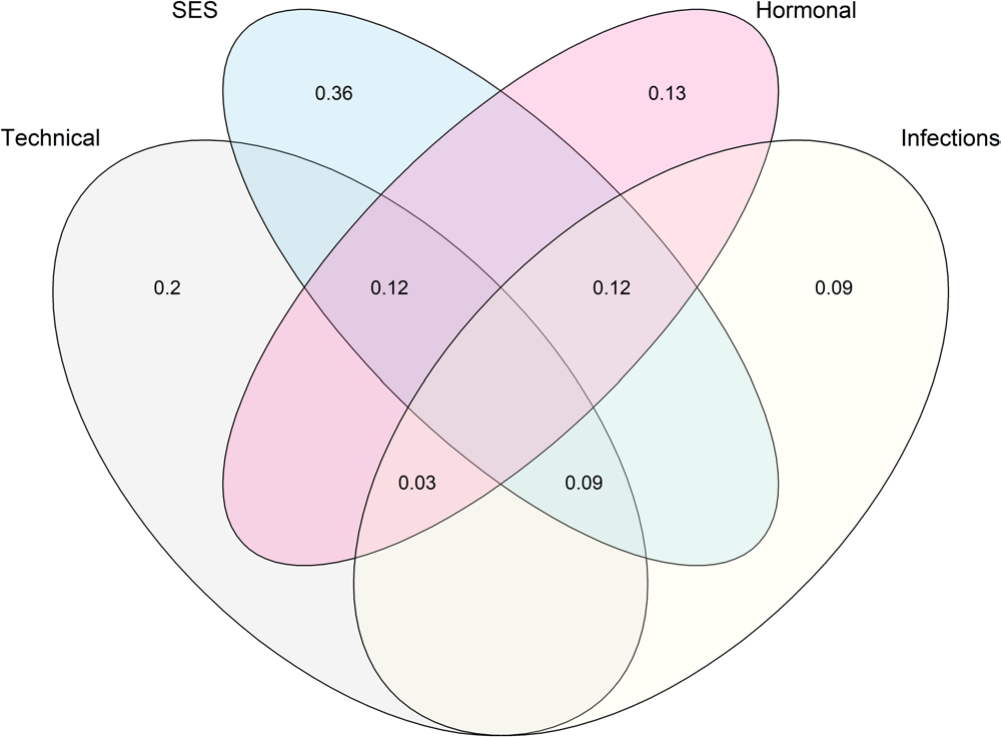
Variance partitioning of microbiota community data with respect to technical variables, socioeconomic factors (SES), estimated hormonal status and infection history. The numbers denote the fraction of the total microbiota variance explained by each of the four variable categories. For the list of individual variables within each group, please see the text.

In order to compare the relative contribution of different types of variables to overall VMB variation, we categorized them to technical or random, socioeconomic status (SES) related, hormonal levels related, and infection and antibiotic use related variables as explained in the methods and used for variance partitioning. The model with SES-related variables explained the highest proportion of the microbiota variation with adjusted R2 = 0.36 (Fig. 3), followed with models for the technical variables (adj. R2 = 0.20), hormonal variables (adj. R2 = 0.13) and infection history (adj. R2 = 0.09).

The individual variables within each of the four categories above were also separately assessed for their correlation with the VMB (Supplementary Table S4). Among the SES-related variables education level was most strongly associated with microbiota variation (R2 = 0.058, p = 0.002, adjusted for age and smoking). Education level correlated positively with L. crispatus (p < 0.05) and inversely with *G*. *vaginalis* (p = 0.03), *A*. *vaginae* (p = 0.006), *Porphyromonas ueonis* (p = 0.03), *Dialister invisus* (p = 0.03) and *Dialister micraerophilus* (p = 0.03). VMB diversity was not associated with education level. Women who were single or divorced had significantly more *G*. *vaginalis*, *A*. *vaginae*, *D*. *invisus*, *Anaerococcus prevotii* and *P*. *timonensis* (p < 0.03) and higher species diversity than cohabiting women (p = 0.02). The number of pregnancies was associated with microbiota variation (R2 = 0.034, p = 0.04), but not with diversity or abundance of any individual species. Smoking was not associated with VMB variation. For the hormone-related variables, the age of the subject was associated with the microbiota variation (R2 = 0.036, p = 0.03), and inversely correlated with *L*. *crispatus* abundance (p = 0.03) but did not associate with species diversity. The phase of the menstrual cycle (follicular vs. luteal) was associated with microbiota variation (R2 = 0.055, p = 0.047), but not with diversity or single species abundance.

The technical or random effects related variables, i.e. DNA concentration, sample weight and sequencing read count, had strong correlation with VMB variation (all p < 0.05). The DNA concentration and sample weight can be interpreted as technical or biological variables, but as our previous work^25^ hints towards the latter (technical variation in the DNA yield was much lower than inter-individual variation), these effects were not studied further. Read count was used as an offset in all statistical models. The infection or antibiotic use -related variables had the smallest contribution to the overall VMB variation, and only the history of recurrent cystitis (n = 7/50) had significant contribution to VMB variation (R2 = 0.032, p = 0.047).

## Discussion

During the past decade, molecular methods, mainly high-throughput sequencing, have greatly expanded our understanding on how the vaginal ecosystem relates to women’s health^1^ as well as to the external^7,10^ and intrinsic factors^2,4,26,27^. The clinical diagnosis of highly common BV and other vaginal infections relies strongly on the microscopic examination of vaginal smears or visual assessment of the vaginal discharge. The correspondence between the microscopic findings and molecular VMB analysis has been previously studied using Nugent score as a reference method^22,28,29^. Here we expand these studies by comparing the light microscopy findings of the Pap smears to VMB composition determined with 16S rRNA amplicon sequencing in asymptomatic women.

These two methods produced highly coherent results regarding the distinction between *Lactobacillus*-dominant versus mixed bacterial communities, but microscopy of the Pap smears could not differentiate *Lactobacillus*-species or identify bacteria in the mixed communities. On the other hand, we found that cytological findings on microscopy provided information that cannot be achieved by sequencing, i.e. inflammation and clue cells which indicate AV and BV.

The diagnosis of BV from Pap smears has a sensitivity of 43.1–59.4% and specificity of 83.3–93.6% compared to the Nugent score, which makes it comparable or better than Amsel’s criteria^30,31^. BV has been associated to a number of key bacteria^32^, of which *Atopobium*, *Prevotella*, *Porhyromonas*, *Peptostreptococcus*, *Mobiluncus* and *Sneathia* were associated with BV in our study. However, *Gardnerella* and *Mageeibacillus indolicus* (formerly known as BV associated bacteria 3, BVAB3) were only associated with LBG III, but not with BV. We did not detect BVAB1–2, *Mycoplasma* or *Ureaplasma* in our cohort. Only four (8%) women had signs of BV in our cohort, likely reflecting the asymptomatic nature of our study population. As we only used clue cells to diagnose BV, it cannot be excluded that the *Gardnerella*-dominant women classified as having ‘mixed bacteria’, actually had BV. Also other molecular studies have shown that BV-associated bacteria are common in women with intermediate Nugent scores^33,34^, which is comparable to our ‘mixed bacteria’-group. The microscopy findings from Pap smears correlated also with sequencing results regarding decreased *Lactobacillus*-dominance, which together with increased diversity has been suggested to be sufficient marker to determine BV in most women^23^. In any case, our approach was not to address the diagnosis of BV, but rather to characterize the normal variation of VMB based on microscopic and phylogenetic analyses. The only reportable factor of the bacterial features of Pap smears according to current Bethesda classification^35^ is the presence of clue cells or *Actinomyces*. According to our results, LBG could be easily incorporated as an additional feature to this classification. Whether LBG is a more descriptive marker for clinically relevant alterations of VMB composition including BV, than the presence of clue cells, cannot be evaluated among asymptomatic women. However, our results indicate that this subject warrants further studies.

The slightly modified AV-score correlated with the depletion of lactobacilli and *G*. *vaginalis* in our cohort, but aerobic *Escherichia coli* or *cocci* traditionally associated with AV^36^ were not detected. However, our cohort did not include any cases with high AV-score typically indicating severe vaginitis and thus, no further conclusions can be drawn. Our modified AV-score for Pap smears did not detect toxic leucocytes as well as the original score based on wet mounts^19^, but should be otherwise comparable.

We found a positive relationship between cytolysis and *L*. *crispatus*. In cytolysis the cytoplasm of intermediate epithelial cell is lysed by lactobacilli, and intracellular glycogen is released from the cell to support their growth. Cytolysis can be considered a physiological process, but excessive cytolysis can result to a condition that has similar symptoms to vulvovaginal candidiasis or another vaginitis. However, it can be is easily differentiated based on acidic pH and high counts of lactobacilli, and lack of yeast hyphae and cells^37^. Due to the asymptomatic nature of our cohort, we consider the observed cytolysis a physiological phenomenon, and the positive association between cytolysis and *L*. *crispatus* reflects rather the favourable growth conditions for *L*. *crispatus* than *L*. *crispatus* causing cytolysis. This hypothesis lends support from a study where the vaginal fluid of women with cytolytic vaginitis had elevated levels of L-lactate, that is produced by other *Lactobacillus* species than the D-lactate producing *L*. *crispatus*^38^.

Apart from our pilot study on 10 women^25^, this is the first study investigating the VMB of Finnish women, and one of the first reports from Nordic countries^39–42^. Similarly to other Caucasian women^1^, the majority (82%) of women in our cohort had *Lactobacillus*-dominated VMB with *L*. *iners* being the dominant bacterium in 38% of the samples. The role of *L*. *iners* is controversial as it is present in VMB both in healthy and dysbiotic states^3^. *L*. *crispatus* was the second most common dominant *Lactobacillus* (34% of samples) followed by *L*. *gasseri*, *L*. *jensenii*, and *L*. *acidophilus* (4%, 4% and 2% of samples, respectively).

Another finding in our study was the association between educational level and the composition of the VMB. Women with higher education had more often *Lactobacillus*-dominated VMB. Previously, education has been associated to BV^43^ and also to VMB composition by Ding *et al*. from the Human Microbiome Project (HMP) cohort^44^. In a recent study, Noyes *et al*.^8^ showed associations between the VMB and demographic factors using Bayesian Network approach. Socioeconomic differences, and specifically the level of education, have also been associated to variation in the gut microbiota^45,46^.

As discussed by Bowyer *et al*. in the TwinsUK study^46^ (N = 1672), socioeconomic factors may influence the human microbiome via different mechanism, involving differences in behavioral (e.g. diet and social contacts) and physiological factors (e.g. stress and health status). Due to the limited characterization of our general population cohort, we could not test if differences e.g. on sexual behavior or hygiene practices associated to the VMB. Hence, identification of the specific factors that mediate the effect of socioeconomic status warrants further studies. The same question about the mediating factors remains open regarding our finding that single or divorced women were more likely to have non-*Lactobacillus* dominant VMB than married or cohabiting women.

In Finland, the participation rate in cervical cancer screening is high (69%^47^), and our study cohort hence represents a cross-section of the population that is ethnically, and even genetically very homogenous. Furthermore, income inequality in Finland is low compared to many other western countries^48^, suggesting that the socio-economic differences within the participants were fairly small. Together with the previous data, our findings highlight the importance of including socio-economic factors as co-variables in the human microbiota studies and suggest that lifestyle changes may provide a robust and attainable approach to modify the vaginal microbiota to support women’s health.

## Methods

### Participants and clinical data

The population based Finnish cervical cancer screening program invites every woman from 30 to 60 years of age with 5-year interval to participate. Some municipalities invite also 25- and 65-year-old women. We sampled 50 non-pregnant, native Finnish (Caucasian) women aged 25 to 45 years attending to Pap smear screening at HUSLAB laboratory in Helsinki. The study was approved by the ethical committee of The Hospital District of Helsinki and Uusimaa and Helsinki region hospital district (21/13/03/03/2014) and performed in accordance with the principles of the Helsinki Declaration. All participants signed an informed consent. All samples were collected in May 2016. The exclusion criteria were vaginal intercourse within 48 hours, pregnancy, previous hysterectomy and inability to tell or remember the time of last menstrual period. All participants signed an informed consent and filled a background questionnaire at the site of sampling. The questionnaire included questions about gynecological history, sexual habits, previous infections, antibiotic and probiotic use, smoking, use of contraceptives, relationship status, and educational status.

### Sample collection and processing

For VMB sampling, sterile flocked swabs (FLOQSwabs, Copan spa, Italy) were rotated on right fornix of the vagina in speculum exam, a collection method validated in our previous work^25^. Speculum was lubricated with sterile saline if needed as in the routine Pap smear protocol at HUSLab laboratory and the Pap smear (two wooden spatulas and a cervical brush) was taken after VMB sampling. Tips of the swabs were severed to 1.5 mL Eppendorf tubes that were frozen in −20 °C right after sampling. The VMB samples were moved to −80 °C freezer within one week.

### Analysis of bacteria in the pap smear

The Pap smears were analyzed with microscopic examinations (phase-contrast, 100*x* and 400*x* magnification). The aim of the microscopy was to extract as many bacteria related features from the smear as possible, not to set a clinical diagnosis. To provide a reproducible classification we used three different classification methods that have been described in the literature:

1. The signs of infection or vaginosis traditionally reported in Pap smears in Finland:
  - Presence of squamous epithelial cells coated with bacteria ie. clue cells as a sign for bacterial vaginosis (BV)^35^.
  - Alterations in typical rod-shaped bacterial flora, presence of coccobacilli-type bacteria, without clue cells, classified here as ‘mixed bacteria’.
  - Only typical rod-shaped bacteria (if visible), classified here as ‘normal’.
2. The *Lactobacillus* grade (LBG) was given with following criteria: LBG I corresponds to normal flora with *Lactobacillus* morphotypes alone, LBG IIA is *Lactobacillus*-dominated, but other morphotypes are present, LBG IIB is dominated by other morphotypes, and LBG III is considered abnormal and lacks *Lactobacillus* morphotypes (21). In addition, lactobacilli were assessed quantitatively.
3. Additionally, a modified aerobic vaginitis (AV) score was calculated using previously described criteria for wet mount samples^19^. The presence of cytolysis, yeast cells/hyphae and blood were also reported. The smears were evaluated by two experienced clinicians (PN, AV) without knowledge of the 16S RNA sequencing results.

### DNA extraction

Bacterial DNA was extracted from the swabs using a previously described bead beating method^25^ with the following modifications: The swabs were vortexed in 0.5 ml of sterile ice-cold PBS, of which 175 *μ*L was combined with 235 *μ*L of RBB lysis buffer (500 mM NaCl, 50 mM Tris-HCl (pH 8.0), 50 mM EDTA, 4% SDS) in a bead beating tube. The samples were bead beaten using a FastPrep-24 instrument at 5.5 m/s (MP Biomedicals, Inc., USA) with 0.1 mm zirconium-silica beads (Biospec Products, Bartlesville, OK, USA) for 1 min. Samples were then heated at +95 °C for 15 min with shaking 400 rpm and centrifuged at room temperature for 5 min at 13 000 rpm. The supernatant (200 *μ*L) was used for DNA extraction with KingFisher Flex automated purification system (ThermoFisher Scientific, USA) and Ambion Magma Total Nucleic Acid Isolation Kit (Life Technologies, USA) using MagMAX Pathogen High Vol Duo program. DNA was quantified using Quanti-iT Pico Green dsDNA Assay (Invitrogen, San Diego, CA, USA). An aliquot of the DNA extract was sent to Karolinska Institutet, Sweden for Human papillomavirus (HPV) genotyping^49^.

### Sequencing of 16S rRNA gene amplicons

Sample preparation for sequencing of the hypervariable V3-V4 regions of the 16S rRNA gene was performed according to the modified protocol by Illumina^50^. Amplification of the 16S rRNA gene fragment (primers 341F 5′-CCTACGGGNGGCWGCAG-3′ and 785Rev 5′-GACTACHVGGGTATCTAATCC-3′) and barcoding primers from Kozich *et al*.^51^ were performed in a single reaction. The PCR reaction comprised of 1 ng/*μ*L template, 1X Phusion High-Fidelity PCR Master Mix (Thermo Scientific, Waltham, MA, USA), 0.25 *μ*M V3-V4 locus specific primers and 0.375 *μ*M dual-index primers. The PCR was run under the following settings: 98 °C for 30 s, 27 cycles of 98 °C for 10 s, 62 °C for 30 s, 72 °C for 15 s and finally 10 min at 72 °C. The PCR clean-up was performed with AMPure XP beads (Beckman Coulter, Copenhagen, Denmark) and confirmation of the right size of the target (ca. 640 base pairs including adapters) was performed on a Bioanalyzer DNA 1000 chip (Agilent Technology, Santa Clara, CA, USA). The pooled libraries were sequenced at the sequencing unit of the Institute for Molecular Medicine Finland (FIMM), Helsinki, Finland with an Illumina HiSeq 2500 sequencer using HiSeq Rapid SBS Kit v2 (2 × 250 bases).

### Sequence preprocessing and analysis

We got 2,872,763 raw sequence pairs from sequencing. The sequence pairs were merged with Illumina utils version 1.4.2 (available at https://github.com/meren/illumina-utils) using “iu-merge-pairs” command^52^. The merging with zero mismatch in the merging region resulted in 2,284,759 reads and enforced Q30 minimum sequencing quality score resulting 1,431,955 reads with an average length of 455 (min 440, max 465), average 15,517 reads per sample (min 1,151, max 53,498). The merged gene sequences were then partitioned using Minimum Entropy Decomposition (MED) that provides unsupervised classification of reads to MED-nodes using Shannon entropy^53,54^. The MED run resulted 1283 MED-nodes. The parameters used for MED can be found in Supplementary Table S5. The 1283 MED-node representative sequences were annotated using BLASTN^55^ to gain species level taxonomy (NCBI 16S database accessed 20 May 2017). To validate the results, we also used R package mare^56^ where taxonomic annotation relies on USEARCH^57^. By using 400nt long merged reads with minimum abundance of 0.001 and annotating the resulting dereplicated and filtered reads with Ribosomal Database Project (RDP) database Training Set 16 (release11) we got essentially identical results for the abundant species (data not shown). Hence, although the accuracy of species-level taxonomic annotation is limited with 16S rRNA gene amplicon data^58^, we were confident to use species-level data due to reassuring annotation statistics from BLASTN (Supplementary Table S1) and identification of the same typical vaginal bacteria with two fully independent methods. As all study subjects were treated equally in respect of species prediction, it is reasonable to assume that the possible species prediction errors have negligible effect on our results excluding the species names.

### Statistical analysis

Statistical analysis was done in R software using packages vegan^59,60^ for calculation of species diversity (inverse Simpson), permutational ANOVA (vegan’s adonis function), and MASS^61^ for generalized linear models using negative binomial distribution with mare package’s^56^ functions GroupTest and CovariateTest for species-wise comparisons between the microscopy- and questionnaire-based grouping presented in the text. The statistical models of mare functions use sample read count as an offset and p-values are corrected for false discovery rate (FDR; Benjamini-Hochberg^62^). Associations between individual background variables and the microbiota were analyzed with permutational ANOVA for the overall microbiota variation, with regression models for negative binomial distributed data for individual bacteria, and ANOVA for the species diversity. Chi-square test and chi-square-test for trend were used for the analysis of categorical data versus gross microbiota variation (IBM SPSS Statistics 22.0; IBM Corp., Armonk, NY). To quantify the contribution of different factors to variation in the VMB composition, we used variance partitioning for *β*-diversity quantified by Bray-Curtis dissimilarity^63^ with varpart function of the vegan package. We grouped variables to four categories: technical or random, socioeconomic status (SES) related, hormonal levels related, and infection- and antibiotic use related. Technical/random variables included DNA concentration, sample weight, recent intercourse, sampling date and sequencing read count. The SES variables in the model were education, marital status, number of pregnancies, working status, smoking, alcohol use and number of sexual partners (lifetime and recent). Estimated hormonal status included period day, phase of the menstrual cycle, contraceptive use and age. The infection history included recent and lifetime antibiotic use, recurrent cystitis, dental infections, history of BV or yeast infection, sexually transmitted infections, severe systemic infections and probiotic use.

## Supplementary information

Supplementary Tables
