# Supplementary material for: Vaginal Microbiota Composition Correlates Between Pap Smear Microscopy and Next Generation Sequencing and Associates to Socioeconomic Status
Source: Sci Rep. 2019 May 23;9:7750. doi: 10.1038/s41598-019-44157-8 (PMC6533281; doi:10.1038/s41598-019-44157-8)
Supplement: Supplementary file 1 — Supplementary Tables [file 41598_2019_44157_MOESM1_ESM.pdf]

Supplementary Information

Vaginal Microbiota Composition Correlates Between Pap Smear Microscopy and Next Generation Sequencing and Associates to Socioeconomic Status

Seppo Virtanen, Tiina Rantsi, Anni Virtanen, Kaisa Kervinen, Pekka Nieminen, Ilkka Kalliala, and Anne Salonen

SUPPLEMENTARY TABLE S1

Bacterial species identified in the 16S rRNA gene sequencing data.

Phylum	Class	Order	Family	Genus	Species	Total counts	Percentage of identical matches	Expect value	NCBI 16S rRNA database hit
Actinobacteria	Actinobacteria	Actinomycetales	Actinomycetaceae	*Mobiluncus*	*curtisii*	188	100	0	*Mobiluncus curtisii* strain V125 16S ribosomal RNA gene, complete sequence
Actinobacteria	Actinobacteria	Actinomycetales	Actinomycetaceae	*Mobiluncus*	*mulieris*	2891	99.562 - 100	0	*Mobiluncus mulieris* strain CCUG 20071 16S ribosomal RNA gene, partial sequence
Actinobacteria	Actinobacteria	Bifidobacteriales	Bifidobacteriaceae	*Gardnerella*	*vaginalis*	48834	98.214 - 100	0	*Gardnerella vaginalis* strain ATCC 14018 16S ribosomal RNA gene, partial sequence
Actinobacteria	Coriobacteriia	Coriobacteriales	Atopobiaceae	*Atopobium*	*vaginae*	29477	98.43 - 100	0	*Atopobium vaginae* strain DSM 15829 16S ribosomal RNA gene, partial sequence
Actinobacteria	Coriobacteriia	Eggerthellales	Eggerthellaceae	*Adlercreutzia*	*equolifaciens*	1214	94.533 - 94.989	0	*Adlercreutzia equolifaciens* strain JCM 14793 16S ribosomal RNA gene, partial sequence
Actinobacteria	Coriobacteriia	Eggerthellales	Eggerthellaceae	*Asaccharobacter*	*celatus*	502	94.533 - 94.761	0	*Asaccharobacter celatus* strain do03 16S ribosomal RNA gene, partial sequence
Bacteroidetes	Bacteroidia	Bacteroidales	Porphyromonadaceae	*Porphyromonas*	*asaccharolytica*	398	99.566	0	*Porphyromonas asaccharolytica* strain ATCC 25260 16S ribosomal RNA gene, partial sequence
Bacteroidetes	Bacteroidia	Bacteroidales	Porphyromonadaceae	*Porphyromonas*	*uenonis*	205	99.566	0	*Porphyromonas uenonis* strain JCM 13868 16S ribosomal RNA gene, partial sequence
Bacteroidetes	Bacteroidia	Bacteroidales	Prevotellaceae	*Prevotella*	*bivia*	362	100	0	*Prevotella bivia* strain JCM 6331 16S ribosomal RNA gene, partial sequence
Bacteroidetes	Bacteroidia	Bacteroidales	Prevotellaceae	*Prevotella*	*buccalis*	255	97.174	0	*Prevotella buccalis* strain JCM 12246 16S ribosomal RNA gene, partial sequence
Bacteroidetes	Bacteroidia	Bacteroidales	Prevotellaceae	*Prevotella*	*disiens*	3197	99.348 - 100	0	*Prevotella disiens* strain JCM 6334 16S ribosomal RNA gene, partial sequence
Bacteroidetes	Bacteroidia	Bacteroidales	Prevotellaceae	*Prevotella*	*salivae*	159	93.492	0	*Prevotella salivae* strain EPSA11 16S ribosomal RNA gene, partial sequence
Bacteroidetes	Bacteroidia	Bacteroidales	Prevotellaceae	*Prevotella*	*timonensis*	7928	99.13 - 100	0	*Prevotella timonensis* strain 4401737 16S ribosomal RNA gene, partial sequence
Firmicutes	Bacilli	Lactobacillales	Lactobacillaceae	*Lactobacillus*	*acidophilus*	9905	99.14 - 100	0	*Lactobacillus acidophilus* strain VPI 6032 16S ribosomal RNA gene, partial sequence
Firmicutes	Bacilli	Lactobacillales	Lactobacillaceae	*Lactobacillus*	*crispatus*	290631	98.925 - 100	0	*Lactobacillus crispatus* strain ATCC 33820 16S ribosomal RNA gene, partial sequence
Firmicutes	Bacilli	Lactobacillales	Lactobacillaceae	*Lactobacillus*	*fornicalis*	12728	99.14 - 100	0	*Lactobacillus fornicalis* strain TV1018 16S ribosomal RNA gene, partial sequence
Firmicutes	Bacilli	Lactobacillales	Lactobacillaceae	*Lactobacillus*	*gallinarum*	1068	99.355 - 99.57	0	*Lactobacillus gallinarum* strain ATCC 33199 16S ribosomal RNA gene, partial sequence
Firmicutes	Bacilli	Lactobacillales	Lactobacillaceae	*Lactobacillus*	*gasseri*	10610	99.14 - 100	0	*Lactobacillus gasseri* strain ATCC 33323 16S ribosomal RNA gene, complete sequence
Firmicutes	Bacilli	Lactobacillales	Lactobacillaceae	*Lactobacillus*	*gasseri*	7645	99.14 - 99.785	0	*Lactobacillus gasseri* strain CIP 102991 16S ribosomal RNA gene, partial sequence
Firmicutes	Bacilli	Lactobacillales	Lactobacillaceae	*Lactobacillus*	*iners*	243588	98.925 - 100	0	*Lactobacillus iners* strain DSM 13335 16S ribosomal RNA gene, partial sequence
Firmicutes	Bacilli	Lactobacillales	Lactobacillaceae	*Lactobacillus*	*jensenii*	27678	99.14 - 100	0	*Lactobacillus jensenii* strain ATCC 25258 16S ribosomal RNA gene, partial sequence
Firmicutes	Bacilli	Lactobacillales	Lactobacillaceae	*Lactobacillus*	*rhamnosus*	266	100	0	*Lactobacillus rhamnosus* strain NBRC 3425 16S ribosomal RNA gene, partial sequence
Firmicutes	Bacilli	Lactobacillales	Streptococcaceae	*Streptococcus*	*agalactiae*	2197	99.355 - 100	0	*Streptococcus agalactiae* strain JCM 5671 16S ribosomal RNA gene, partial sequence
Firmicutes	Clostridia	Clostridiales	Lachnospiraceae	*Lachnobacterium*	*bovis*	46489	91.176 - 92.081	8.49e-178 - 3.98e-171	*Lachnobacterium bovis* strain LRC 5382 16S ribosomal RNA gene, partial sequence
Firmicutes	Clostridia	Clostridiales	Lachnospiraceae	*Moryella*	*indoligenes*	325	95.682	0	*Moryella indoligenes* strain AIP 220.04 16S ribosomal RNA gene, partial sequence
Firmicutes	Clostridia	Clostridiales	Peptostreptococcaceae	*Peptostreptococcus*	*anaerobius*	2256	99.544 - 100	0	*Peptostreptococcus anaerobius* strain NCTC 11460 16S ribosomal RNA gene, partial sequence
Firmicutes	Clostridia	Clostridiales	Peptostreptococcaceae	*Peptostreptococcus*	*stomatis*	246	99.772	0	*Peptostreptococcus stomatis* strain W2278 16S ribosomal RNA gene, partial sequence
Firmicutes	Clostridia	Clostridiales	Ruminococcaceae	*Mageeibacillus*	*indolicus*	2607	90.828 - 100	0 - 2.41e-168	*Mageeibacillus indolicus* strain 0009-5 16S ribosomal RNA, partial sequence
Firmicutes	Negativicutes	Veillonellales	Veillonellaceae	*Dialister*	*invisus*	2975	94.444 - 94.872	0	*Dialister invisus* strain JCM 17566 16S ribosomal RNA gene, partial sequence
Firmicutes	Negativicutes	Veillonellales	Veillonellaceae	*Dialister*	*micraerophilus*	283	100	0	*Dialister micraerophilus* strain JCM 17567 16S ribosomal RNA gene, partial sequence
Firmicutes	Negativicutes	Veillonellales	Veillonellaceae	*Megasphaera*	*cerevisiae*	3962	92.077 - 92.719	0	*Megasphaera cerevisiae* strain DSM 20462 16S ribosomal RNA gene, partial sequence
Firmicutes	Negativicutes	Veillonellales	Veillonellaceae	*Megasphaera*	*elsdenii*	721	93.348 - 93.562	0	*Megasphaera elsdenii* strain DSM 20460 16S ribosomal RNA gene, partial sequence
Firmicutes	Tissierellia	Tissierellales	Peptoniphilaceae	*Anaerococcus*	*prevotii*	347	100	0	*Anaerococcus prevotii* strain DSM 20548 16S ribosomal RNA gene, complete sequence
Firmicutes	Tissierellia	Tissierellales	Peptoniphilaceae	*Parvimonas*	*micra*	1753	97.267 - 97.722	0	*Parvimonas micra* strain JCM 12970 16S ribosomal RNA gene, partial sequence
Firmicutes	Tissierellia	Tissierellales	Peptoniphilaceae	*Peptoniphilus*	*lacrimalis*	192	100	0	*Peptoniphilus lacrimalis* strain CCUG 31350 16S ribosomal RNA gene, partial sequence
Fusobacteria	Fusobacteriia	Fusobacteriales	Fusobacteriaceae	*Fusobacterium*	*animalis*	1552	99.323 - 99.549	0	*Fusobacterium nucleatum subsp. animalis* strain NCTC 12276 16S ribosomal RNA gene, partial sequence
Fusobacteria	Fusobacteriia	Fusobacteriales	Leptotrichiaceae	*Sneathia*	*sanguinegens*	9571	97.291 - 100	0	*Sneathia sanguinegens* strain CCUG 41628 16S ribosomal RNA gene, complete sequence
Proteobacteria	Gammaproteobacteria	Pseudomonadales	Pseudomonadaceae	*Pseudomonas*	*veronii*	669	100	0	*Pseudomonas veronii* strain CIP 104663 16S ribosomal RNA gene, complete sequence

SUPPLEMENTARY TABLE S2

Comparison of microscopy-based Pap smear features and sequencing results.

Feature	Sequencing result	Estimate	p-value
***Lactobacillus* grade (III vs. I)**			
	Species diversity	1.87	< 0.0001
	Permutational ANOVA	0.22	< 0.0001
**Single species differences**			
	*Mobiluncus mulieris**	10.93	< 0.0001
	*Lachnobacterium bovis**	7.93	< 0.0001
	Gardnerella vaginalis	6.27	< 0.0001
	Adlercreutzia equolifaciens	2.53	0.0001
	Mageeibacillus indolicus	2.47	0.0019
	Dialister micraerophilus	2.29	< 0.0001
	Megasphaera cerevisiae	2.27	0.0087
	Peptoniphilus lacrimalis	1.98	< 0.0001
	Moryella indoligenes	1.41	0.0074
	Lactobacillus acidophilus	-1.22	0.0262
	Lactobacillus iners	-4.39	< 0.0001
	*Lactobacillus crispatus**	-7.38	< 0.0001
**Clue cells (YES vs. NO)**			
	Species diversity	3.09	< 0.0001
	Permutational ANOVA	0.08	< 0.0001
**Single species differences**			
	*Mobiluncus mulieris**	11.12	< 0.0001
	*Lachnobacterium bovis**	7.43	< 0.0001
	Prevotella timonensis	6.00	< 0.0001
	Atopobium vaginae	5.87	< 0.0001
	Sneathia sanguinegens	4.91	< 0.0001
	Dialister invisus	3.24	0.0130
	Prevotella disiens	3.21	0.0014
	Adlercreutzia equolifaciens	2.99	0.0004
	Peptostreptococcus anaerobius	2.94	0.0014
	Mobiluncus curtisii	2.87	< 0.0001
	Moryella indoligenes	2.14	0.0006
	Peptoniphilus lacrimalis	1.89	0.0044
	Anaerococcus prevotii	1.57	0.0417
	Dialister micraerophilus	1.44	0.0130
	Pseudomonas veronii	-1.64	0.0174
	Lactobacillus acidophilus	-2.06	0.0195
	Lactobacillus fornicalis	-2.41	0.0368
	*Lactobacillus iners**	-6.16	< 0.0001
	*Lactobacillus crispatus**	-7.44	< 0.0001

**Cytolysis (YES vs. NO)**			
	Species diversity	-0.90	0.0142
	Permutational ANOVA	0.14	< 0.0001
**Single species differences**			
	Lactobacillus crispatus	5.16	< 0.0001
	Lactobacillus gallinarum	2.37	< 0.0001
	Lactobacillus iners	-2.62	0.0469
	Lactobacillus fornicalis	-3.51	< 0.0001
	Dialister invisus	-4.48	0.0001
	Gardnerella vaginalis	-5.66	< 0.0001
	Atopobium vaginae	-6.89	< 0.0001
**AV score (0-6 scale)**			
	Species diversity	1.26	< 0.0001
	Permutational ANOVA	0.12	< 0.0001
**Single species differences**			
	Gardnerella vaginalis	1.19	< 0.0001
	Peptoniphilus lacrimalis	0.49	0.0035
	Dialister micraerophilus	0.37	0.0182
	Fusobacterium nucleatum	0.28	0.0161
	Porphyromonas uenonis	0.26	0.0149
	Prevotella salivae	0.21	0.0387
	*Lactobacillus crispatus**	-1.04	0.0003
**Mixed bacteria (YES vs. NO)**			
	Species diversity	1.35	0.0002
	Permutational ANOVA	0.12	< 0.0001
**Single species differences**			
	Sneathia sanguinegens	6.13	< 0.0001
	Gardnerella vaginalis	5.78	< 0.0001
	Atopobium vaginae	4.67	0.0002
	Dialister invisus	4.54	< 0.0001
	Anaerococcus prevotii	2.40	< 0.0001
	Megasphaera cerevisiae	2.25	0.0029
	Mageeibacillus indolicus (BVAB3)	2.20	0.0018
	Peptoniphilus lacrimalis	1.89	0.0042
	Dialister micraerophilus	1.66	0.0002
	Moryella indoligenes	1.01	0.0306
	Prevotella bivia	0.98	0.0462

**Leucocytes (amount, 4 level scale)**			
	Species diversity	-0.08	0.6000
	Permutational ANOVA	0.03	0.1000
**Single species differences**			
	Pseudomonas veronii	0.49	0.0485
***Lactobacillus* (amount, 4 level scale)**			
	Species diversity	-0.48	0.0004
	Permutational ANOVA	0.13	< 0.0001
**Single species differences**			
	Lactobacillus fornicalis	1.43	0.0017
	Lactobacillus crispatus	1.27	< 0.0001
	Lactobacillus gallinarum	0.61	0.0033
	Prevotella bivia	-0.38	0.0325
	Peptoniphilus lacrimalis	-0.63	0.0033
	Dialister micraerophilus	-1.03	0.0017
	Mobiluncus curtisii	-1.09	0.0131
	Peptostreptococcus stomatis	-1.31	< 0.0001
	Peptostreptococcus anaerobius	-1.44	< 0.0001
	Gardnerella vaginalis	-1.57	0.0005
	Dialister invisus	-1.60	< 0.0001
	Anaerococcus prevotii	-1.61	0.0008
	Atopobium vaginae	-1.68	0.0006
	Streptococcus agalactiae	-1.80	< 0.0001
	Prevotella timonensis	-2.04	< 0.0001
	Sneathia sanguinegens	-2.31	< 0.0001
	Parvimonas micra	-3.30	0.0003
**Yeast (YES vs. NO)**			
	Species diversity	-0.19	0.8205
	Permutational ANOVA	0.01	0.8500

Statistics

Diversity statistics were calculated with ANOVA for group comparison and with GLM for scales. Permutational ANOVA was calculated with the adonis-function of the vegan-package and the estimate is the R2 value. Differences in species abundances were calculated with CovariateTest and GroupTest functions of the mare-package. The p-values from the mare-package were adjusted with FDR method (Benjamin-Hochberg). *For species with highly zero-inflated abundance we used zeroinfl-function of the pscl-package to provide estimates and p-values which however should be interpreted with caution.

SUPPLEMENTARY TABLE S3

Patient characteristics compared to coarse vaginal microbiota from 16S gene sequencing. One patient was excluded from contraception analysis due to use of tamoxifen medication.

	***Lactobacillus crispatus* - dominated**			***Lactobacillus iners*- dominated**			**Non-*Lactobacillus*- dominated**		
**Characteristics**	**+**	**-**	***p-value***	**+**	**-**	***p-value***	**+**	**-**	***p-value***
**Total number (%)**	17 (34.0)	33 (66.0)		20 (40.0)	40 (60.0)		9 (18.0)	41 (82.0)	
**Age, years**[a]			**0.02**			0.61			0.09
Mean (SD, range)	30.3 (6.5, 25-45)	35.2 (6.8, 25-45)		34.0 (6.8, 25-45)	33.2(7.3, 25-45)		37.2 (6.8, 25-45)	32.3(6.9, 25-45)	
Median	30.0	35.0		35.0	30.0		40.0	35.0	
**Smoking status, n (%)**[b]			0.35			0.90			0.47
Current smoker	4 (23.5)	13 (39.4)		7 (35.0)	10 (33.3)		4 (44.4)	13 (31.7)	
Non-smoker	13 (76.5)	20 (60.6)		13 (65.0)	20 (66.7)		5 (55.6)	28 (68.3)	
**Stable relationship (>6 months)**[b]			0.49			0.90			0.06
Yes	12 (70.6)	20 (60.6)		13 (65.0)	19 (63.3)		3 (33.3)	29 (70.7)	
No	5 (29.4)	13 (39.4)		7 (35.0)	11 (36.7)		6 (66.7)	12 (29.3)	
**Marital status, n (%)**[b]			0.37			1.00			**0.03**
Married / cohabiting	11 (64.7)	17 (51.5)		11 (55.0)	17 (56.7)		2 (22.2)	26 (63.4)	
Divorced / single	6 (35.3)	16 (48.5)		9 (45.0)	13 (43.3)		7 (77.8)	15 (36.6)	
**Number of sex partners in lifetime, n (%)**[c]			0.37			0.23			0.35
1-3	7 (41.2)	4 (12.1)		1 (5.0)	10 (33.3)		1 (11.1)	10 (24.4)	
4-10	2 (11.8)	12 (36.4)		8 (40.0)	6 (20.0)		2 (22.2)	12 (29.3)	
10-20	4 (23.5)	11 (33.3)		7 (35.0)	8 (26.7)		4 (44.4)	11 (26.8)	
>20	4 (23.5)	6 (18.2)		4 (20.0)	6 (20.0)		2 (22.2)	8 (19.5)	
**Number of sex partners in past 12 months, n (%)**[c]			0.59			0.09			0.43
0-1	15 (88.2)	24 (72.7)		13 (65.0)	26 (86.7)		7 (77.8)	32 (78.0)	
2-3	0	5 (15.2)		3 (15.0)	2 (6.7)		2 (22.2)	3 (7.3)	
>3	2 (12.5)	4 (12.1)		4 (20.0)	2 (6.7)		0	6 (14.6)	
**Education, n (%)**[c]			**0.02**			0.38			**<0.001**
Comprehensive school	0	1 (3.0)		0	1 (3.3)		1 (11.1)	0	
Vocational upper secondary education	0	7 (21.2)		2 (10.0)	5 (16.7)		4 (44.4)	3 (7.3)	
Technical college	0	2 (6.1)		1 (5.0)	1 (3.3)		1 (11.1)	1 (2.4)	
General upper secondary education	2 (11.8)	3 (9.1)		2 (10.0)	3 (10.0)		1 (11.1)	4 (9.8)	
High degree education (university or polytechnic)	15 (88.2)	20 (60.6)		15 (75.0)	20 (66.7)		2 (22.2)	33 (80.5)	
**Parity, n (%)**[b]			**0.04**			0.90			**0.02**
Nulliparous	8 (47.1)	25 (75.8)		13 (65.0)	20 (66.7)		9 (100.0)	24 (58.5)	
Parous	9 (52.9)	8 (24.2)		7 (35.0)	10 (33.3)		0	17 (100.0)	
**History of fertility treatment, n (%)**[b]			1.00			0.08			**0.03**
Yes	2 (11.8)	3 (9.1)		0	5 (16.7)		3 (33.3)	2 (4.9)	
No	15 (88.2)	30 (90.9)		20 (100.0)	25 (83.3)		6 (66.7)	39 (95.1)	
**Contraception, n (%)**[b]									
Combined contraception			0.12			1.00			0.66
Yes	6 (35.3)	5 (15.6)		4 (20.0)	7 (24.1)		1 (11.1)	10 (25.0)	
No	11 (64.7)	27 (84.4)		16 (80.0)	22 (75.9)		8 (88.9)	30 (75.0)	
Progestin pill			1.00			1.00			1.00
Yes	1 (5.9)	3 (9.4)		2 (10.0)	2 (6.9)		0	4 (10.0)	
No	16 (94.1)	29 (90.6)		18 (90.0)	27 (93.1)		9 (100.0)	36 (90.0)	
Long acting reversible contraception (LARC)			0.70			1.00			0.63
Yes	2 (11.8)	6 (18.8)		3 (15.0)	5 (17.2)		2 (22.2)	6 (15.0)	
No	15 (88.2)	26 (81.3)		17 (85.0)	24 (82.8)		7 (77.8)	34 (85.0)	
Nothing / condom			0.54			0.82			0.47
Yes	8 (47.1)	19 (56.3)		11 (55.0)	15 (51.7)		6 (66.7)	20 (50.0)	
No	9 (52.9)	14 (43.8)		9 (45.0)	14 (48.3)		3 (33.3)	20 (50.0)	
**Use of probiotics, n (%)**[b]			0.14			0.08			1.00
Yes	11 (64.7)	14 (42.4)		7 (35.0)	18 (60.0)		5 (55.6)	20 (48.8)	
No	6 (35.3)	19 (57.6)		13 (65.0)	12 (40.0)		4 (44.4)	21 (51.2)	

[a] P-values calculated using Mann-Whitney U-test
[b] P-values calculated using Chi-squared test
[c] P-values calculated using Chi-squared test for trend

SUPPLEMENTARY TABLE S4

Permutational ANOVA results, assessing the effect of background variables on microbiota variation. Calculated with adonis function of the vegan package. All variables were combined in a single model. Statistically significant p-values are bolded. The variables were grouped to technical related, socioeconomic state (SES) related, hormonal related and infection history related variables. This grouping is somewhat artificial but helps to compare the relative contribution of numerous factors to the microbiota variation.

	Variable	Df	SumsOfSqs	MeanSqs	F.Model	R2	Pr(>F)
**SES**	Education level	1	1.078	1.078	4.010	0.058	**0.002**
	Cohabitation	1	0.302	0.302	1.123	0.016	0.329
	Previous pregnancies	1	0.623	0.623	2.317	0.034	**0.038**
	Working status	1	0.319	0.319	1.185	0.017	0.303
	Smoking	1	0.330	0.330	1.227	0.018	0.277
	Alcohol consumption	1	0.252	0.252	0.939	0.014	0.460
	Sex partners (lifetime)	1	0.430	0.430	1.598	0.023	0.145
	Sex partners (12 months)	1	0.222	0.222	0.824	0.012	0.558
	Stable relationship	1	0.194	0.194	0.723	0.010	0.650
**Hormonal**	Age	1	0.662	0.662	2.464	0.036	**0.030**
	Contraception	6	1.783	0.297	1.105	0.096	0.343
	Menstrual cycle phase	2	1.014	0.507	1.886	0.055	**0.047**
	Period day	1	0.177	0.177	0.658	0.010	0.710
**Technical**	DNA concentration	1	0.978	0.978	3.639	0.053	**0.004**
	Sample weight	1	0.758	0.758	2.819	0.041	**0.015**
	Recent intercourse (48h)	1	0.183	0.183	0.682	0.010	0.704
	Sampling date	5	1.807	0.361	1.344	0.098	0.144
	Sequencing read count	1	0.782	0.782	2.908	0.042	**0.014**
**Infections and antibiotics**	History of tooth infenction	1	0.477	0.477	1.773	0.026	0.103
	History of infection requiring i.v. antibiotics	1	0.375	0.375	1.397	0.020	0.205
	History of Chlamydia	1	0.481	0.481	1.788	0.026	0.101
	Herpes simplex infection	1	0.456	0.456	1.696	0.025	0.117
	Recurrent cystitis	1	0.586	0.586	2.181	0.032	**0.047**
	History of yeast infection	1	0.181	0.181	0.675	0.010	0.695
	History of recurrent yeast infection	1	0.211	0.211	0.784	0.011	0.588
	History of BV	1	0.213	0.213	0.793	0.012	0.585
	Antibiotic use (3 months)	1	0.194	0.194	0.723	0.010	0.650
	Antibiotic use (lifetime)	1	0.560	0.560	2.082	0.030	0.060
	Probiotic use	1	0.204	0.204	0.760	0.011	0.613
	Residuals	10	2.689	0.269		0.145	
	Total	49	18.522			1.000	

SUPPLEMENTARY TABLE S5

Parameters for Minimum Entropy Decomposition (MED).

Min entropy for a component to be picked for decomposition	0.0965
Perform entropy normalization heuristics	True
Max number of discriminants to use for decomposition	12
Min total abundance of oligotype in all samples	0
Min substantive abundance of an oligotype (-M)	50
Maximum variation allowed in each node (-V)	2 nt
Nodes agglomerated based on co-occurence patterns	
Merge homopolymer splits	False
Skip removing outliers	False
Try to relocate outliers	True
